# Enhanced effects of cigarette smoke extract on inflammatory cytokine expression in IL-1β-activated human mast cells were inhibited by Baicalein via regulation of the NF-κB pathway

**DOI:** 10.1186/1476-7961-10-3

**Published:** 2012-02-06

**Authors:** David S Chi, Ta-Chang Lin, Kenton Hall, Tuanzhu Ha, Chuanfu Li, Zong Doa Wu, Thomas Soike, Guha Krishnaswamy

**Affiliations:** 1 Department of Internal Medicine, James H. Quillen College of Medicine, East Tennessee State University, Johnson City, Tennessee 37614, USA; 2 Department of Environmental Engineering, National Cheng Kung University, Tainan, Taiwan; 3 Departmen of Surgery, James H. Quillen College of Medicine, East Tennessee State University, Johnson City, Tennessee 37614, USA

**Keywords:** Mast cell, cigarette smoking, Baicalein, IL-6, IL-8, NF-κB activation, IκBα phosphorylation and degradation

## Abstract

**Background:**

Human mast cells are capable of a wide variety of inflammatory responses and play a vital role in the pathogenesis of inflammatory diseases such as allergy, asthma, and atherosclerosis. We have reported that cigarette smoke extract (CSE) significantly increased IL-6 and IL-8 production in IL-1β-activated human mast cell line (HMC-1). Baicalein (BAI) has anti-inflammatory properties and inhibits IL-1β- and TNF-α-induced inflammatory cytokine production from HMC-1. The goal of the present study was to examine the effect of BAI on IL-6 and IL-8 production from CSE-treated and IL-1β-activated HMC-1.

**Methods:**

Main-stream (Ms) and Side-stream (Ss) cigarette smoke were collected onto fiber filters and extracted in RPMI-1640 medium. Two ml of HMC-1 at 1 × 10^6 ^cells/mL were cultured with CSE in the presence or absence of IL-1β (10 ng/mL) for 24 hrs. A group of HMC-1 cells stimulated with both IL-1β (10 ng/ml) and CSE was also treated with BAI. The expression of IL-6 and IL-8 was assessed by ELISA and RT-PCR. NF-κB activation was measured by electrophoretic mobility shift assay (EMSA) and IκBα degradation by Western blot.

**Results:**

Both Ms and Ss CSE significantly increased IL-6 and IL-8 production (p < 0.001) in IL-1β-activated HMC-1. CSE increased NF-κB activation and decreased cytoplasmic IκBα proteins in IL-1β-activated HMC-1. BAI (1.8 to 30 μM) significantly inhibited production of IL-6 and IL-8 in a dose-dependent manner in IL-1β-activated HMC-1 with the optimal inhibition concentration at 30 μM, which also significantly inhibited the enhancing effect of CSE on IL-6 and IL-8 production in IL-1β-activated HMC-1. BAI inhibited NF-κB activation and increased cytoplasmic IκBα proteins in CSE-treated and IL-1β-activated HMC-1.

**Conclusions:**

Our results showed that CSE significantly increased inflammatory cytokines IL-6 and IL-8 production in IL-1β-activated HMC-1. It may partially explain why cigarette smoke contributes to lung and cardiovascular diseases. BAI inhibited the production of inflammatory cytokines through inhibition of NF-κB activation and IκBα phosphorylation and degradation. This inhibitory effect of BAI on the expression of inflammatory cytokines induced by CSE suggests its usefulness in the development of novel anti-inflammatory therapies.

## Background

Human mast cells, which are associated with allergies, asthma, and atherosclerosis, are multifunctional cells capable of inflammatory responses producing and secreting a wide variety of lipid mediators, histamine, cytokines, and chemokines [1,2]. Mast cells have been implicated in acute and chronic inflammatory responses and in many diseases characterized by inflammation [3]. The fact that mast cells accumulate at sites of inflammation, such as the nasal mucosa of patients with allergic rhinitis [4], the lung smooth muscle of patients with asthma [5], the skin of patients with urticaria [6], and the joints of patients with arthritis [7], illustrates the association of mast cells in these inflammatory diseases [8]. Our previous reviews have summarized the important role mast cells play in allergic, asthmatic, and inflammatory responses, conditions caused by the production of mediators and select inflammatory cytokines [1,2].

Interleukin-6 (IL-6) and interleukin-8 (IL-8) are important inflammatory cytokines that are secreted from activated mast cells. IL-6 is a multifunctional protein. In innate immunity, it stimulates the synthesis of acute-phase proteins by hepatocytes and thus contributes to the systemic effects of inflammation [9]. In adaptive immunity, it stimulates the growth of B cells that have differentiated into antibody producers [10]. IL-8 is a potent neutrophil chemotactic and activating factor. It serves as a chemical signal that attracts neutrophils to the site of inflammation [11]. IL-1β is secreted mainly by macrophages. IL-1β is produced in response to various stimulants, such as bacteria, viruses, and cytokines [12]. Our previous studies have shown IL-1β activated human mast cells produce selected inflammatory cytokines [13,14].

The Centers for Disease Control and Prevention reported that the adverse health effects from cigarette smoking account for an estimated 438,000 deaths or nearly 1 out of every 5 deaths each year in the United States. Cigarette smoking is linked to cancer, cardiovascular disease, respiratory disease, and other adverse effects. Epidemiological studies also show that cigarette smoking increases the risk of atherosclerosis [15]. Unfortunately, the underlying basic mechanisms involved in the processes that lead to diseases induced by cigarette smoke components is not much known. Previously, we have reported that cigarette smoke extract (CSE) significantly increased IL-6 and IL-8 production in IL-1β-activated human mast cell-1 (HMC-1). The first goal of this study was to examine effects and mechanisms of CSE on the expression of inflammatory cytokines in mast cells. Studying mast cell responses to CSE may lead to a greater understanding of cigarette smoke induced diseases.

Baicalein (BAI) is a flavonoid originally isolated from the roots of the traditional Chinese herbal medicine Huangqin, *Scutellaria baicalensis *Georgi. It has been widely employed for many centuries in the traditional Chinese herbal medicine as popular antibacterial, antiviral, and anti-inflammatory agents [16]. Historically, *Scutellaria baicalensis *has been used to treat respiratory tract infection, diarrhea, jaundice, and hepatitis. Recent investigations showed it had broad anti-inflammatory activities. BAI suppressed the LPS-induced production of NO in RAW 264.7 mouse macrophages [17]. It was shown to have potent neuroprotective effect on LPS-induced injury of dopaminergic neurons [18]. Recently, BAI has been shown to inhibit inflammation through inhibition of COX-2 gene expression [19] and to suppress LPS induced degradation of IκBα and activation of NF-κB [20]. Recently, our group has reported that Baicalein inhibits IL-1β- and TNF-α-induced inflammatory cytokine production from human mast cells via regulation of NF-κB pathway [21]. The second goal of this study is to investigate effects and mechanisms of BAI on inflammatory cytokine expressions from CSE treated and IL-1β-activated human mast cells. Our results showed that BAI inhibited the enhanced effects of CSE on expression of inflammatory cytokines through inhibition of NF-κB activation and IκBα phosphorylation and degradation in human mast cells. This inhibitory effect of BAI on the expression of inflammatory cytokines suggests its usefulness in the development of novel anti-inflammatory therapies.

## Methods

### Reagents and cells

The baicalein was purchased from Sigma (St. Louis, MO). The HMC-1 cell line, established from a patient with mast cell leukemia, was graciously provided by Dr. Joseph H. Butterfield (Mayo Clinic, Rochester, MN). IL-1β and ELISA kits of IL-6, and IL-8 were purchased from R&D (Minneapolis, MN). RPMI 1640 media and HEPES were obtained from GibcoBRL (Rockville, MD). 2-mercaptoethanol was purchased from Sigma (St. Louis, MO). Fetal bovine serum was obtained from Atlanta Biologicals (Atlanta, GA). RNA-BEE was purchased from Tel-Test, Inc. (Friendswood, Texas). Gene Amp RNA PCR Core Kit was purchased from Applied Biosystems (Branchburg, NJ).

### Cigarette smoke extract

An ignited cigarette was placed in a 3.1 L bell-shaped glass vessel, the Sidestream smoke (Ss) within the vessel was pumped out of the bell and collected onto quartz fiber filters. Mainstream smoke (Ms) was collected directly from the cigarette onto quartz fiber filters using a puff volume of 35 ml in 2 seconds at a rate of 8 puffs per minute. The filters were weighed before and after smoke collection and the increase in weight was recorded as cigarette smoke weight. The cigarette smoke was extracted from the filters with RPMI 1640 medium to a concentration of 5 mg/ml.

### Cell culture

HMC-1 cells were cultured and maintained in RPMI 1640 media with 5 × 10^-5 ^2-mercaptoethanol, 10 mM HEPES, gentamycin 50 μg/ml, 5 μg/ml insulin, transferrin and sodium selenite, 2 mM L-glutamine, and 5% heat inactivated fetal bovine serum in a 37°C incubator with 5% CO_2_. The cell cultures were maintained in 75 cm^2 ^flasks (Corning) [22].

### Induction of cytokine production

Two ml of HMC-1 mast cells at 1 × 10^6 ^cells/ml concentration were cultured with or without different concentrations of both Ms and Ss cigarette smoke extract in the presence or absence of IL-1β (10 ng/ml) for 24 hrs [13]. A group of HMC-1 cells stimulated with both IL-1β (10 ng/ml) and CSE was also treated with BAI. The cultures were carried out in triplicate. At the end of incubation, supernatants were harvested for measuring IL-6 and IL-8 by ELISA and cell viability and numbers of the culture were analyzed. The cell viability was determined by trypan blue dye exclusion. Trypan blue dye (0.4%) was added to cell samples in a ratio of 1:2.5 and preparations were viewed with a standard light microscope [13]. The ratio of live to dead cells (cell viability) was determined. The cell viabilities of the drug groups and the medium control cultures in this study were ranging from 90 to 98%. BAI, IL-1β, and CSE at the concentrations used in this study appeared to have no toxic effect to the HMC-1 cultures.

### ELISA for cytokine production

Cytokine ELISA was performed for IL-6 and IL-8. ELISA was carried out on cell-free culture supernatants using commercially available ELISA kits, according to manufacturer's instructions as earlier described. Results were analyzed on an ELISA plate reader (Dynatech MR 5000 with supporting software) [13].

### Analysis of cytokine gene expression by RT-PCR

HMC-1 were treated with the appropriate reagents and allowed to incubate at 37°C with 5% CO_2 _for 6 hours before being harvested for RNA. RNA was extracted from HMC-1 (3 × 10^6 ^cells) by the addition of 1 ml of RNA-BEE. After the addition of chloroform and shaking for 1 minute the samples were centrifuged at 12,000 × g for 15 minutes at 4°C to achieve phase separation. Isopropanol was added to the aqueous phase, and the preparation was frozen at -20°C overnight. The following day, the samples were centrifuged at 12,000 × g for 30 minutes at 4°C. The RNA pellet was washed with 1 ml 75% ethanol containing DEPC and allowed to air dry. The pellet was resuspended in DEPC water and quantitated by optical density readings at 260 nm. Reverse Transcriptase Polymer Chain Reaction (RT-PCR) was performed with a Gene Amp RNA PCR Core Kit according to manufacturer's instructions. cDNA was synthesized with murine leukemia virus reverse transcriptase (2.5 U/μl), 10× PCR buffer (500 mM KCl, 100 mM Tris-HCl, pH 8.3), 1 mM each of the nucleotides dATP, dCTP, dGTP and dTTP; RNase inhibitor (1 U/μl), MgCl_2 _(5 mM), and oligo(dT)_16 _(2.5 μM) as a primer. The samples were incubated at 42°C for 20 minutes, 99°C for 20 minutes, and 5°C for 5 minutes in a DNA thermocycler (Perkin-Elmer Corp., Norwalk, CT) for reverse transcription. PCR of cDNA was done with MgCl_2 _(1.8 mM), each of the dNTPs (0.2 mM), AmpliTaq polymerase (1 U/50 μl), and paired cytokine-specific primers (0.2 nM of each primer) to a total volume of 50 μl. Cycles consisted of 1 cycle of 95°C for 2 min, 35 cycles of 95°C for 45 sec, 60°C for 45 sec, and 72°C for 1 min 30 sec, and lastly, 1 cycle of 72°C for 10 min. Ten microliters of the sample were electrophoresed on a 2% agarose gel and stained with ethidium bromide for viewing. Primer sequences used are as follows: HPRT: 5' CGA GAT GTG ATG AAG GAG ATG G 3' and 5' GGA TTA TAC TGC CTG ACC AAG G 3'; IL-6: 5' ATG AAC TCC TTC TCC ACA AGC GC 3' and 5' GAA GAG CCC TCA GGC TGG ACT G 3'; and IL-8: 5' ATG ACT TCC AAG CTG GCC GTG GCT 3' and 5' TCT CAG CCC TCT TCA AAA ACT TCT C 3'. Densitometry was done by normalizing target genes to house keepers using Un-Scan-It Version 5.1 software (Orem, UT) [21].

### NF-κB assay in HMC-1

HMC-1 were stimulated with IL-1β, CSE, and/or BAI for 30 minutes, and then harvested for isolation of nuclear and cytoplasmic proteins according our previously reported method [21]. Nuclear translocation of NF-κB was analyzed by the electrophoretic mobility shift assay (EMSA) using the nuclear proteins [23-26]. Cells were washed with PBS and mixed with one hundred microliters of hypotonic buffer which contains: 10 mM HEPES pH 7.9, 10 mM KCl, 0.1 mM EDTA, 0.1 mM EGTA, 1 mM dithiothreitol (DTT), 0.5 mM phenylmethylsulfonyl fluoride (PMSF), 1 μM aprotinin, 1 μM pepstatin, 14 μM leupeptin, 50 mM NaF, 30 mM β-glycerophosphate, 1 mM Na_3_VO_4_, and 20 mM p-nitrophenyl phosphate. Cells were incubated over ice for 30 minutes and then vortexed after the addition of 6.25 μl of 10% of Nonidet P-40. After 2 minutes of centrifugation at 30,000 × g, supernatants were kept at -80°C while the pellets were collected and vortexed every 20 minutes for 3 hours in 60 ml of a hypertonic salt solution: 20 mM HEPES pH 7.9, 0.4 M NaCl, 1 mM EDTA, 1 mM EGTA, 12 mM DTT, 1 mM PMSF, 1 μM aprotinin, 1 μM pepstatin, 14 μM leupeptin, 50 mM NaF, 30 mM β-glycerophosphate, 1 mM Na_3_VO_4_, and 20 mM p-nitrophenyl phosphate. Nuclear translocation of NF-κB was analyzed by the EMSA using the nuclear fraction. Seven micrograms of nuclear protein were added to 2 ml of binding buffer (Promega, Madison, WI), and 35 fmol of double stranded NF-κB consensus oligonucleotide (5' AGT TGA GGG GAC TTT CCC AGG C 3') (Promega, Madison, WI) end labeled with γ-P32 ATP (Amersham Biosciences, Piscataway, NJ). The samples were incubated at room temperature for 20 minutes and run on a 5% nondenaturing polyacrylamide gel for 2 hours. A supershift assay using antibodies to P65 and P50 was performed to confirm NF-κB binding specificity as previously described [23-26].

### Western blot analysis for IκBα

Cytoplasmic proteins (40 μg) were mixed with 2x SDS sample buffer, heated at 95°C for 5 min, and separated by SDS-polyacrylamide (12.5%) gel electrophoresis [24,27]. The separated proteins were transferred onto Hybond enhanced chemiluminescence membranes (Amersham) and then incubated with an appropriate rabbit primary antibody [IκBα antibody (Santa Cruz Biotechnology) or phosphorylated IκBα antibody (New England Biolabs)] in Tris-buffered saline - 0.05% Tween 20 containing 5% nonfat dry milk for 1 - 2 hours at room temperature. After they were washed three times in Tris-buffered saline - 0.05% Tween 20, the membranes were incubated with peroxidase-conjugated goat anti-rabbit IgG (Sigma Chemical) for 1 hour at room temperature. After three washes in PBS, the conjugated peroxidase was visualized by enhanced chemiluminescence according to the manufacturer's instructions (Amersham). The protein signals of IκBα were quantified by scanning densitometry (Genomic Solutions).

### Statistical analysis of the data

All experiments were done in triplicate. The data were analyzed by Student's two-tailed *t*-test using Statistica software (StatSoft, Inc., Tulsa, OK). All data were reported as means ± SE. A *p*-value of less than 0.05 was considered significant.

## Results

### CSE increased IL-6 and IL-8 production in IL-1β-activated mast cells

**Figure 1:**
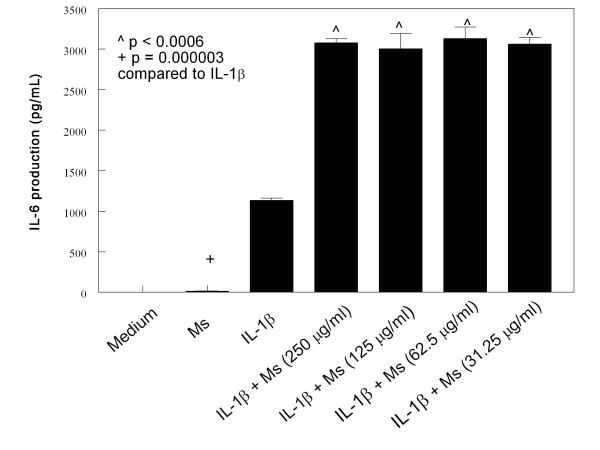
**Effects of Mainstream cigarette smoke extract (Ms) on IL-6 production from IL-1β-activated HMC-1 cells**. To each well of a 6-well culture plate, two ml of HMC-1 (1 × 10^6 ^cells/ml) were cultured alone (Medium), or in the presence of IL-1β (10 ng/ml), and the combinations of IL-1β with different concentrations of Ms for 24 hrs in triplicate. Supernatants were harvested for measuring IL-6 by ELISA.

**Figure 2:**
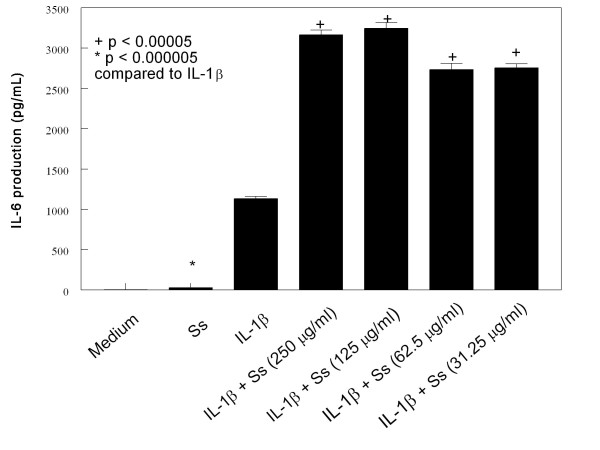
**Effects of Sidestream cigarette smoke extract (Ss) on IL-6 production from IL-1β-activated HMC-1 cells**. To each well of a 6-well culture plate, two ml of HMC-1 (1 × 10^6 ^cells/ml) were cultured alone (Medium), or in the presence of IL-1β (10 ng/ml), and the combinations of IL-1β with different concentrations of Ss for 24 hrs in triplicate. Supernatants were harvested for measuring IL-6 by ELISA.

**Figure 3:**
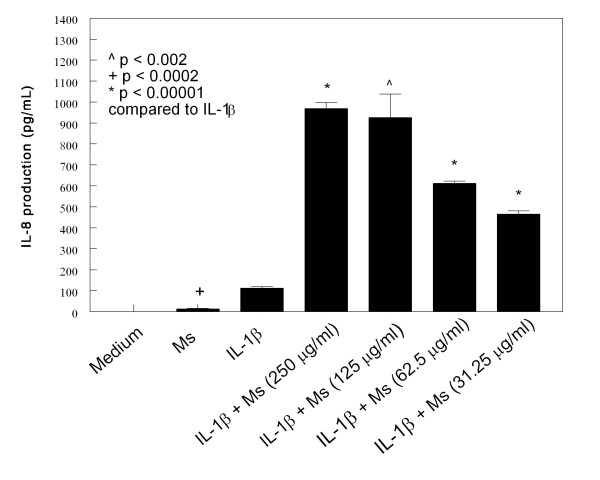
**Effects of Mainstream cigarette smoke extract (Ms) on IL-8 production from IL-1β-activated HMC-1 cells**. To each well of a 6-well culture plate, two ml of HMC-1 (1 × 10^6 ^cells/ml) were cultured alone (Medium), or in the presence of IL-1β (10 ng/ml), and the combinations of IL-1β with different concentrations of Ms for 24 hrs in triplicate. Supernatants were harvested for measuring IL-8 by ELISA.

**Figure 4:**
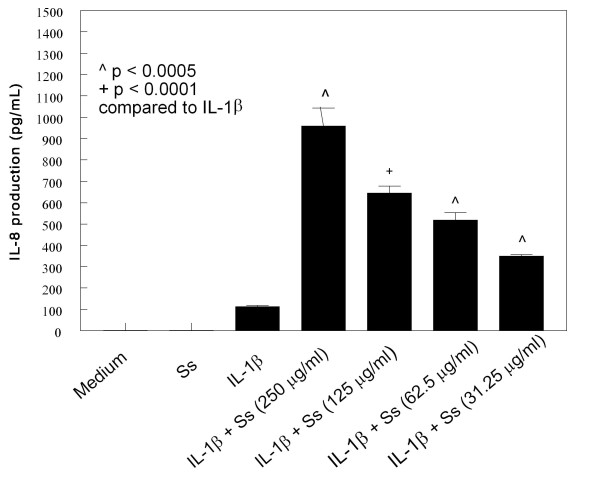
**Effects of Sidestream cigarette smoke extract (Ss) on IL-8 production from IL-1β-activated HMC-1 cells**. To each well of a 6-well culture plate, two ml of HMC-1 (1 × 10^6 ^cells/ml) were cultured alone (Medium), or in the presence of IL-1β (10 ng/ml), and the combinations of IL-1β with different concentrations of Ss for 24 hrs in triplicate. Supernatants were harvested for measuring IL-8 by ELISA.

HMC-1 cells were cultured with IL-1β (10 μg/mL) and various concentration of CSE for 24 hours. The medium alone did not induce IL-6 and IL-8 production in HMC-1 cells (Figures 1, 2, 3 and 4). As the previous report, IL-1β at 10 ng/mL concentration markedly induced IL-6 and IL-8 production from HMC-1, while the mainstream (Ms) and sidestream (Ss) CSE either did not or only produced trace of cytokines (Figures 1, 2, 3 and 4). The effect of Ms CSE on production of IL-6 was shown in Figure 1. The Ms CSE at 31.25 to 250 μg/mL concentrations significantly increased IL-6 production in IL-1β-activated HMC-1 cells. The Ss CSE also increased production of IL-6 in IL-1β-activated HMC-1 cells (Figure 2). Similarly, both Ms and Ss CSE increased production of IL-8 in a dose-dependent fashion in IL-1β-activated HMC-1 cells (Figures 3 and 4).

### BAI inhibits the enhancing effects of CSE on IL-6 and IL-8 production in IL-1β-activated mast cells

**Figure 5:**
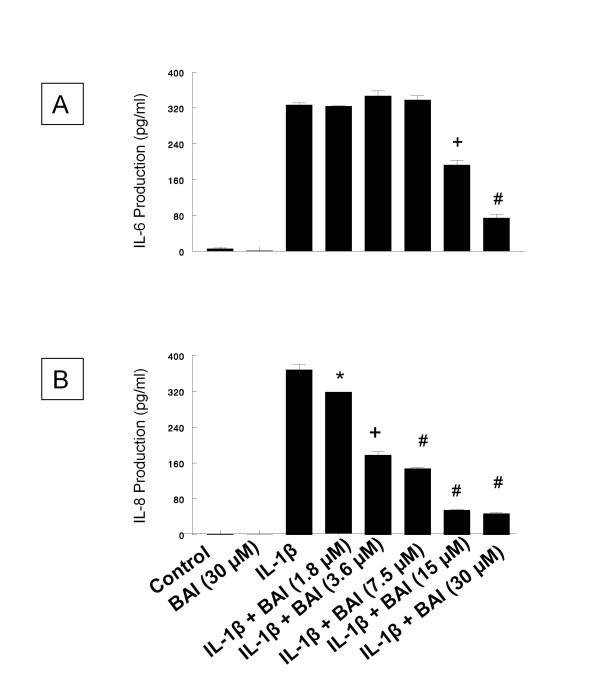
**Effects of Baicalein (BAI) on production of IL-6 (A) and IL-8 (B) from IL-1β-activated HMC-1 cells**. (*, +, and # indicate p < .05, < .0005, and < .00005, respectively, when compared with the IL-1β-treated group)

The effect of BAI on production of IL-6 and IL-8 from IL-1β-activated HMC-1 cells was studied. BAI at concentrations of 1.8, 3.6, 7.5, 15, and 30 μM have been proved to be non-toxic to HMC-1 [28]. Two mL of HMC-1 at 1 × 10^6 ^cells/mL were cultured with the above mentioned concentrations of BAI in the presence or absence of IL-1β (10 ng/mL) for 24 hrs. The culture supernatants were collected and assayed for cytokines by ELISA. BAI alone did not induce cytokine production from HMC-1. However, BAI at 15 and 30 μM concentrations significantly decreased the IL-6 production to 192.7 ± 18.7 and 74.6 ± 14.6 pg/mL, respectively (p < 0.0005 and p < 0.00005, respectively) (Figure 5A). BAI at all tested concentrations (1.8 to 30 μM) significantly decreased the IL-1β-induced IL-8 production, in a dose-dependent manner, to 316.4 ± 1.3, 177.4 ± 13.2, 147.6 ± 5.4, 54.9 ± 3.3, and 46.9 ± 4.4 pg/mL, respectively (p < 0.05 for 1.8 μM, p < 0.0005 for 3.6 μM, and p < 0.00005 for all the rest) (Figure 5B). Since BAI at 30 μM was the most effective concentration in inhibition of cytokine production in IL-1β-activated HMC-1, we decided to only use this concentration in experiments with CSE-treated HMC-1.

**Figure 6:**
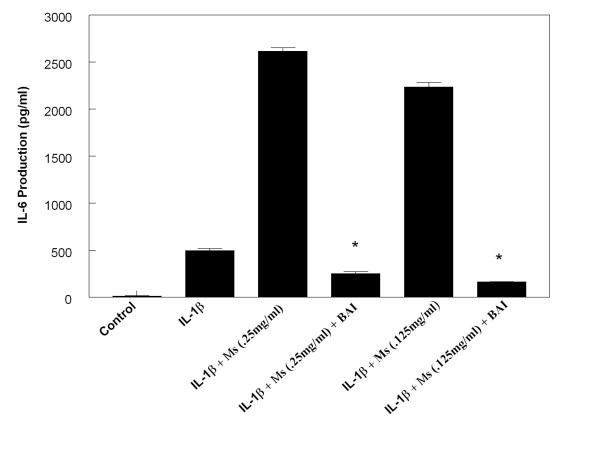
**Effects of Baicalein (BAI, 30 μM) on production of IL-6 from Mainstream cigarette smoke extract (Ms)-treated and IL-1β-activated HMC-1**. (* indicates p < .000005 when compared with the corresponding IL-1β + Ms groups)

**Figure 7:**
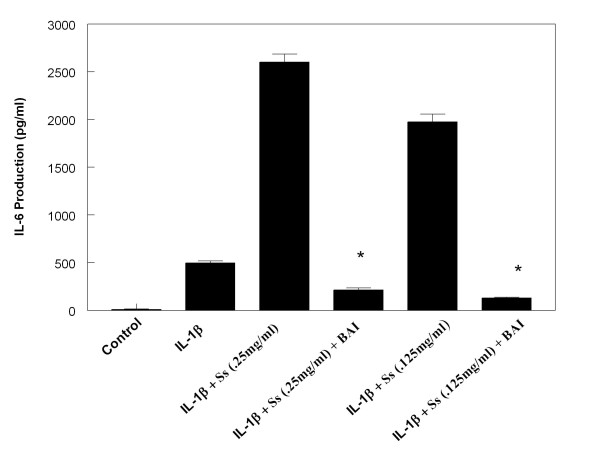
**Effects of Baicalein (BAI, 30 μM) on production of IL-6 from Sidestream cigarette smoke extract (Ss)-treated and IL-1β-activated HMC-1**. (* indicates p < .00005 when compared with the corresponding IL-1β + Ss groups)

**Figure 8:**
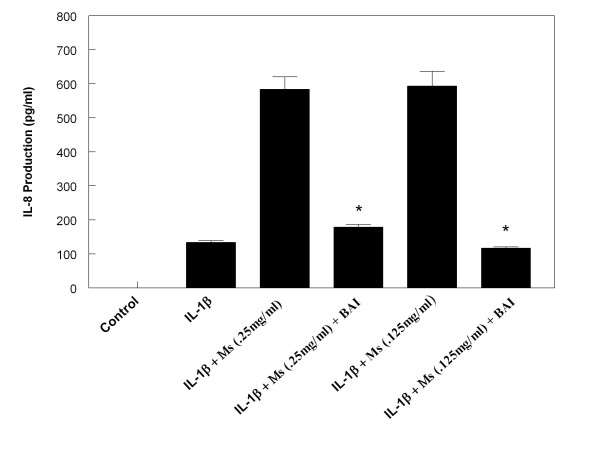
**Effects of Baicalein (BAI, 30 μM) on production of IL-8 from Mainstream cigarette smoke extract (Ms)-treated and IL-1β-activated HMC-1**. (* indicates p < .0005 when compared with the corresponding IL-1β + Ms groups).

**Figure 9:**
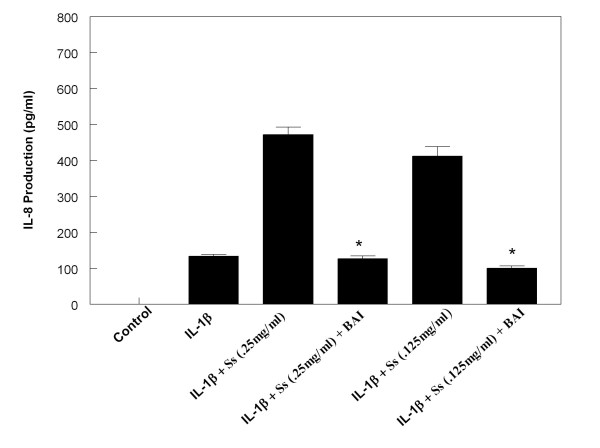
**Effects of Baicalein (BAI, 30 μM) on production of IL-8 from Sidestream cigarette smoke extract (Ss)-treated and IL-1β-activated HMC-1**. (* indicates p < .0005 when compared with the corresponding IL-1β + Ss groups)

In order to investigate the effect of BAI on the enhancing effects CSE has on IL-6 and IL-8 production in IL-1β-activated mast cells, we treated the HMC-1 cells with or without IL-1β and CSE in the presence or absence of BAI (30 μM). BAI significantly inhibited the enhancing effect of both Ms and Ss CSE on production of IL-6 in IL-1β-activated HMC-1 (Figures 6 and 7). Similarly, BAI also significantly inhibited the enhancing effect of both Ms and Ss CSE on production of IL-8 in IL-1β-activated HMC-1 (Figures 8 and 9).

### Effects of BAI on IL-6 and IL-8 gene expressions in CSE-treated and IL-1β-activated mast cells

**Figure 10:**
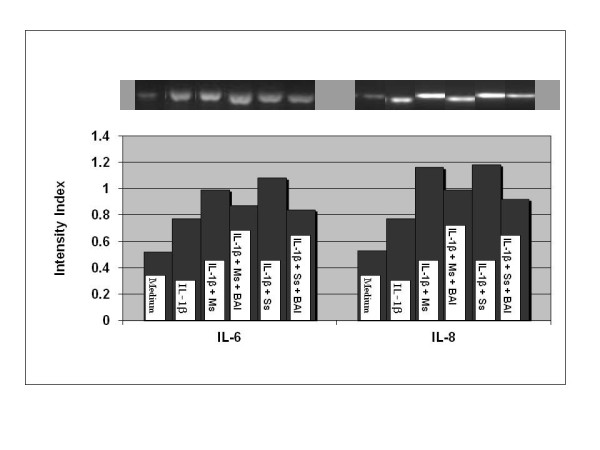
**RT-PCR analysis of effects of BAI on the gene expression of IL-6 and IL-8 in cigarette smoke extracts (CSE: Mainstream, Ms, and Sidestream, Ss)-treated and IL-1β- activated HMC-1 cells**. HMC-1 were treated with IL-1β (10 μg/mL) and CSE in the presence or absence of BAI (30 μM) for 6 hours and harvested for transcriptional analysis via RT-PCR. The intensities of the cytokine and house keeping gene (HPRT) bands were measured by densitometry, and the ratio of the cytokine to the house keeping gene was calculated and assigned as the intensity index. IL-1β-treated HMC-1 increased IL-6 and IL-8 mRNA transcription. In the presence of Ms (0.125 mg/mL) or Ss (0.125 mg/mL) CSE, the expression of IL-6 and IL-8 was increased further. However, in the addition of BAI, the expression of IL-6 and IL-8 was remarkably decreased.

To study effects of BAI on inflammatory cytokine gene expression, the experiments were performed using IL-1β-activated HMC-1. HMC-1 were treated with IL-1β (10 μg/mL) and CSE in the presence or absence of BAI (30 μM) for 6 hours and harvested for transcriptional analysis via RT-PCR. IL-1β-treated HMC-1 increased IL-6 and IL-8 mRNA transcription (Figure 10). The intensities of the cytokine and house keeping gene (HPRT) bands were measured by densitometry, and the ratio of the cytokine to the house keeping gene was calculated and assigned as the intensity index. In the presence of Ms (0.125 mg/mL) or Ss (0.125 mg/mL) CSE, the expression of IL-6 and IL-8 was increased. However, in the present of BAI, the expression of IL-6 and IL-8 was remarkably decreased (Figure 10).

### Role of NF-kB activation in the inhibitory effect of BAI on inflammatory cytokine production from CSE-treated and IL-1β-activated mast cells

**Figure 11:**
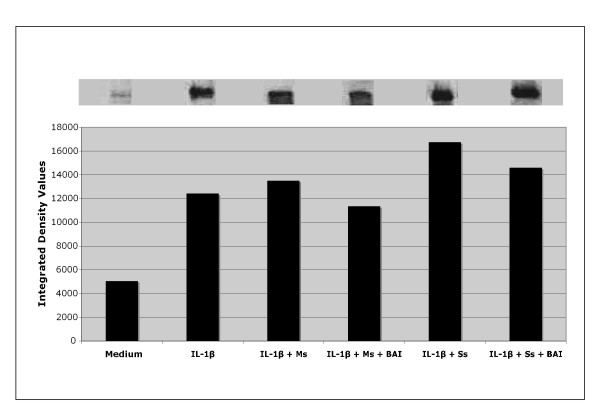
**Effects of BAI on NF-κB translocation in IL-1β-activated and cigarette smoke extract (CSE: Mainstream, Ms, and Sidestream, Ss)-treated HMC-1 cells**. HMC-1 were treated with IL-1β (10 μg/mL) and CSE (0.125 mg/mL) in the presence or absence of BAI (30 μM) for 24 hours. NF-κB translocation was analysed by a shift in oligonucleotide binding in EMSA gels. Densitometric analysis of NF-κB was expressed as integrated intensity.

NF-κB is an important transcription factor that mediates the transcription of many proinflammmatory cytokine genes [29,30]. In order to study the role that NF-κB plays in the inhibitory effect of BAI on inflammatory cytokine production, NF-κB activation was analyzed in HMC-1 cultured with CSE and IL-1β (10 μg/mL) in the presence or absence of BAI (30 μM). NF-κB translocation, as seen by a shift in oligonucleotide binding in EMSA gels, was increased in the IL-1β-activated HMC-1 (Figure 11). With the addition of Ms or Ss CSE (0.125 mg/mL) treatment, NF-κB translocation was even higher than that of the only IL-1β-treated group (Figure 11). In the presence of BAI, NF-κB translocation was decreased when compared to that of the IL-1β and CSE-activated HMC-1 (Figure 11).

### Role of IkBα proteins in the inhibitory effect of BAI on inflammatory cytokine production from CSE-treated and IL-1β-activated mast cells

**Figure 12:**
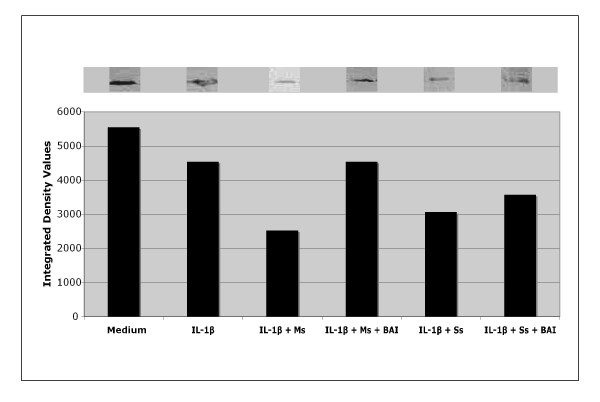
**Effects of BAI on IκBα proteins levels in cytoplasm of IL-1β-activated and cigarette smoke extract (CSE: Mainstream, Ms, and Sidestream, Ss)-treated HMC-1 cells**. HMC-1 were treated with IL-1β (10 μg/mL) and CSE (0.125 mg/mL) in the presence or absence of BAI (30 μM) for 24 hours. Cytoplasmic extracts were prepared from each sample, and levels of IκBα proteins were analysed by Western blot. Densitometric analysis of NF-κB was expressed as integrated intensity.

The activation of NF-κB requires phosphorylation and proteolytic degradation of the inhibitory protein IκBα [31]. To determine whether the inhibitory activity of BAI is due to its effect on IκBα phosphorylation and degradation, we used Western blot analysis to examine the cytoplasmic levels of IκBα in HMC-1 after treatment with Ms or Ss CSE (both 0.125 mg/mL) and IL-1β (10 μg/mL) in the presence or absence of BAI (30 μM). The data showed that in the presence of IL-1β, the IκBα protein levels were decreased in HMC-1 (Figure 12). In cell treated with IL-1β and Ms or Ss CSE, the IκBα protein levels were further decreased when compared to that of the only IL-1β-activated group (Figure 12). With the addition of BAI, the IκBα protein levels were markedly increased when compared to that of the IL-1β- and CSE-activated HMC-1 (Figure 12).

## Discussion

Inflammatory cytokines are important factors in chronic inflammation, allergy, asthma, atherogenesis, and autoimmune diseases. Human mast cells play an integral role in the inflammatory response by accumulating at sites of inflammation and mediating the production of inflammatory cytokines, such as IL-6 and IL-8 [32].

IL-6 promotes the formation of acute phase proteins in the liver during the acute phase response and initiates B-cell transformation into plasma cells. IL-8 serves as a chemo-attractant for neutrophils, macrophages, and T-cells.

The expression of various inflammatory cytokines is regulated by transcription factors. The activation of the NF-κB transcription plays an important role in inflammation through its ability to induce the transcription of proinflammatory genes [33]. The NF-κB exists as a homo- or hetero-dimer comprised of 65-kDa (Rel A or p65) and 50 kDa (p50) DNA-binding proteins, and is a ubiquitously expressed transcription factor that exists in a latent state in the cytoplasm bound to inhibitory proteins, IκBα. Following an activation stimulus, IκBα undergoes degradation, allowing free NF-κB to localize to the nucleus where it binds to specific recognition elements in the promoter regions of various genes. This can then initiate transcription of cytokines.

Cigarette smoking is linked to cancer, cardiovascular disease, respiratory disease, and other adverse effects such as atherosclerosis [15]. However, the underlying basic mechanisms involved in the processes that lead to diseases induced by cigarette smoke components is not much known. Previously, we have reported that cigarette smoke extract (CSE) significantly increased IL-6 and IL-8 production in IL-1β-activated human mast cell-1 (HMC-1). In this study, we aimed to examine effects and mechanisms of CSE on the expression of inflammatory cytokines in mast cells.

Our results showed that both Main- and Side-stream CSE increase IL-6 and IL-8 production in IL-1β-activated mast cells (Figures 1, 2, 3, and 4). Moreover, RT-PCR analysis of the gene expression of the inflammatory cytokines, IL-6 and IL-8, was markedly increased in smoke extract-treated and IL-1β-activated HMC-1 (Figure 10). This suggests that the ability of CSE to increase cytokine production is through the increase of cytokine mRNA transcription. Furthermore, CSE increased NF-κB binding activity (Figure 11) and decreased IκBα proteins in the cytoplasm of IL-1β-activated mast cells (Figure 12). These results suggest CSE enhances the NF-κB activation via inhibition of IκBα phosphorylation and degradation.

In spite of advances in the pharmacological management of the above mentioned chronic inflammatory diseases and symptoms, to discover effective, alternative anti-inflammatory reagents is still needed. Several Chinese herbal medicines have anti-bacterial and viral properties and been used for treatment of chronic inflammation. Previously, we have screened several Chinese herbal medicines and found that the compound Baicalein (BAI) isolated from Huangqin (*Scutellaria baicalensis *Georgi) has a great inhibitory effect on the production of IL-6 from IL-1β-activated HMC-1 in a dose dependent fashion [28]. In this study we further investigate inhibitory effects and mechanisms of BAI on inflammatory cytokine expression from IL-1β-activated and CSE-treated human mast cells.

The results showed that BAI (1.8 to 30 μM) significantly inhibited production of IL-6 and IL-8 in a dose-dependent manner in IL-1β-activated HMC-1 (Figure 5). Since BAI 30 μM was the most effective concentration, we only used this dose to treat smoke extract-treated and IL-1β-activated HMC-1 cells. We found BAI also significantly inhibited production of IL-6 and IL-8 in smoke extract-treated and IL-1β-activated HMC-1 (Figures 6, 7, 8, and 9). The cell viabilities of the drug groups and medium control cultures ranged from 90 to 98%. Thus, this inhibitory effect appears not due to the toxic effect of BAI on HMC-1 cells. Moreover, the gene expression, analyzed by RT-PCR, of these inflammatory cytokines was markedly decreased in smoke extract-treated and IL-1β-activated HMC-1 (Figure 10) when BAI was present. This suggests that the inhibitory effect of BAI on cytokine productions is through the decrease of cytokine mRNA transcription.

Previously, glucocorticoids that have frequently been used for the treatment of inflammatory diseases, allergy, and autoimmune diseases were thought to suppress NF-κB activation. Glucocorticoids induce the transcription of IκBα, resulting in an enlarged IκBα pool, and therefore reduced active NF-κB in the nucleus [34]. Additionally, 12-lipoxygenase (12-LOX) has been implicated as a mediator of inflammation, atherosclerosis, and cancer [35-37]. Several *in vitro *studies have suggested 12/15-LOX products to be co-activators of peroxisomal proliferator activating-receptors (PPAR), regulators of cytokine generation, and modulators of gene expression related to inflammation resolution. The dampening effect of PPAR on inflammation is via their inhibitory activity on expression of NF-κB [38-40]. As BAI is known as a 12-LOX inhibitor, we speculated the mechanism by which BAI inhibited inflammatory cytokines was through the NF-κB/IκBα pathway. Therefore, we analyzed NF-κB activation and examined the cytoplasmic levels of IκBα in HMC-1 after treatment with IL-1β and CSE in the presence or absence of BAI. Our data showed BAI decreased NF-κB binding activity (Figure 11) and increased IκBα proteins in the cytoplasm of IL-1β-activated and CSE-treated mast cells (Figure 12). The results suggest BAI inhibits the NF-κB activation via inhibition of IκBα phosphorylation and degradation.

In recent studies, an important flavonoid, quercetin, has been reported to exert a strong inhibitory effect on the production of IL-6, MCP-1, and histidine decarboxylase (HDC) mRNA transcription from mast cells [41-43]. Our results confirmed that BAI, as a flavonoid, could also strongly inhibit production of inflammatory cytokines of IL-6 and IL-8 from activated mast cells through the decrease of mRNA transcription. Ultimately it is hoped that BAI will be a possible candidate for future development of novel anti-inflammatory therapies.

## Conclusions

Cigarette smoke extract (CSE) significantly increased IL-6 and IL-8 production in IL-1β-activated human mast cell-1 (HMC-1). CSE derived increases in cytokine production is due to the increase of cytokine mRNA transcription. Furthermore, CSE increased NF-κB binding activity and decreased IκBα proteins in the cytoplasm of IL-1β-activated mast cells. The results may partially explain why cigarette smoke contributes to lung and cardiovascular diseases.

In searching for effective drugs to treat inflammatory related diseases, we found Baicalein from Chinese herbal medicine possessed a strong inhibitory effect on production of selected inflammatory cytokines from human mast cells. The inhibitory mechanism appears to be due to inhibition of NF-κB activation pathway and IκBα phosphorylation and degradation. This inhibitory effect of Baicalein on the expression of inflammatory cytokines indicates its usefulness in the development of novel anti-inflammatory therapies.

## List of abbreviations

BAI: Baicalein; CSE: cigarette smoke extract; Ms: mainstream smoke; Ss: Sidestream smoke; EMSA: electrophoretic mobility shift assay; HMC-1: human mast cell-1; IκBα: inhibitor of κB alpha; MCP-1: monocyte chemotactic protein 1; NF-κB: nuclear factor-kappa B

## Competing interests

The authors declare that they have no competing interests.

## Authors' contributions

DSC designed and conducted experiments, analysed data, and wrote the manuscript. TCL and ZDW collected cigarette smoke extract and performed reference search. KH conducted experiments and proofread manuscript. TH and CL contributed to the experiments of EMSA and Western blot. TS conducted experiments and analysed data. GK oversaw research. The authors have had the opportunities to both read and approve the final manuscript.
